# Ectopic activation of WNT signaling in human embryonal carcinoma cells and its effects in short- and long-term *in vitro* culture

**DOI:** 10.1038/s41598-019-48396-7

**Published:** 2019-08-15

**Authors:** Yaser Atlasi, Rebecca T. van Dorsten, Andrea Sacchetti, Rosalie Joosten, J. Wolter Oosterhuis, Leendert H. J. Looijenga, Riccardo Fodde

**Affiliations:** 1 Dept. of Pathology, Erasmus MC Cancer Institute, Erasmus University Medical Center, Rotterdam, The Netherlands; 2 Present Address: Faculty of science, Radboud University, Nijmegen, The Netherlands; 3 Present Address: Witwatersrand University, Johannesburg, South Africa; 4 Present Address: Princess Maxima Center for Pediatric Oncology, Utrecht, The Netherlands

**Keywords:** Pluripotent stem cells, Germ cell tumours

## Abstract

Human embryonal carcinoma (EC) cells comprise the pluripotent stem cells of malignant non-seminomatous germ cell tumors (GCTs) and represent the malignant counterpart of embryonic stem cells (ESCs). WNT/β-catenin signaling has been implicated in regulating adult and embryonic stem cells although its role in EC cells is less investigated. Here, we studied WNT signaling in a panel of representative pluripotent and nullipotent human EC cell lines. We found that EC cell lines show distinct levels of intrinsic WNT signaling and respond differently to ectopic WNT activation. Short-term activation of WNT signaling induced a differentiation-response in the pluripotent EC cells (NT2 and NCCIT) whereas the nullipotent EC cells (TERA1 and 2102Ep) were refractory and maintained high levels of OCT4 and SSEA4 expression. Long-term activation of WNT signaling in NCCIT and, to a lesser extent, TERA1 cells led to (re)gain of OCT4 expression and a switch from SSEA4 to SSEA1 surface antigens ultimately resulting in OCT4^+^/SSEA4^−^/SSEA1^+^ profile. Cisplatin treatment indicated that the OCT4^+^/SSEA4^−^/SSEA1^+^ NCCIT cells became more resistant to chemotherapy treatment. Our findings are of particular interest for the GCT and ES cell biology and shed light on the role of WNT signaling in human EC cells.

## Introduction

Type II malignant GCTs, also known as (testicular) Germ Cell Cancers (TGCCs), are the most common malignancies in Caucasian young adults and include Seminomas (SE) and Non-Seminoma (NS) tumors^1,2^. Embryonal Carcinomas (EC) are the pluripotent stem cell compartment of NS and can differentiate to somatic cells (to generate teratomas) or extraembryonic tissues (i.e. yolk sac tumor [YSTs] and choriocarcinoma)^3^. A large body of evidence shows striking similarities between human EC cells and human embryonic stem cells (derived from the pre-implantation embryo), thus suggesting that EC cells might represent the malignant counterpart of hESCs^4–7^.

WNT signaling is involved in different stages of embryonic development and controls, among others, the self-renewal of adult and embryonic stem cells. The multi-protein ‘destruction complex’ consisting of, among others, the glycogen synthase kinase 3 beta (GSK3β), earmarks β-catenin for ubiquitination ultimately leading to its proteolytic degradation. Activation of WNT signaling results in the destabilization of this complex and increased β-catenin levels in the cytoplasm and nucleus. Here β-catenin acts as a transcriptional co-factor to regulate a broad spectrum of downstream target genes^8^. In mouse ES and EC cells, WNT signaling supports self-renewal and represses multi-lineage differentiation^9–13^. In human ESCs, however, ectopic activation of WNT signaling supports only the short-term self-renewal^14–17^ and its inhibition does not affect hESC maintenance thus suggesting that endogenous WNT signaling does not play a major role in long term self-renewal of hESCs^13,18^. In TGCCs, global gene expression profiling together with β-catenin immunohistochemical analysis revealed enrichment of WNT signaling activity in EC and YSTs^19–21^. The functional impact of WNT signaling in hEC cells, however, is less investigated.

Here, we ectopically activated WNT signaling in different hEC cell lines and evaluated its subsequent effects in short- and long-term cultures. Our findings describe the divergent effects of WNT signaling in hEC lines and report new intermediate states that can be captured in these cells.

## Results

### hEC cell lines display different levels of endogenous WNT signaling

**Figure 1:**
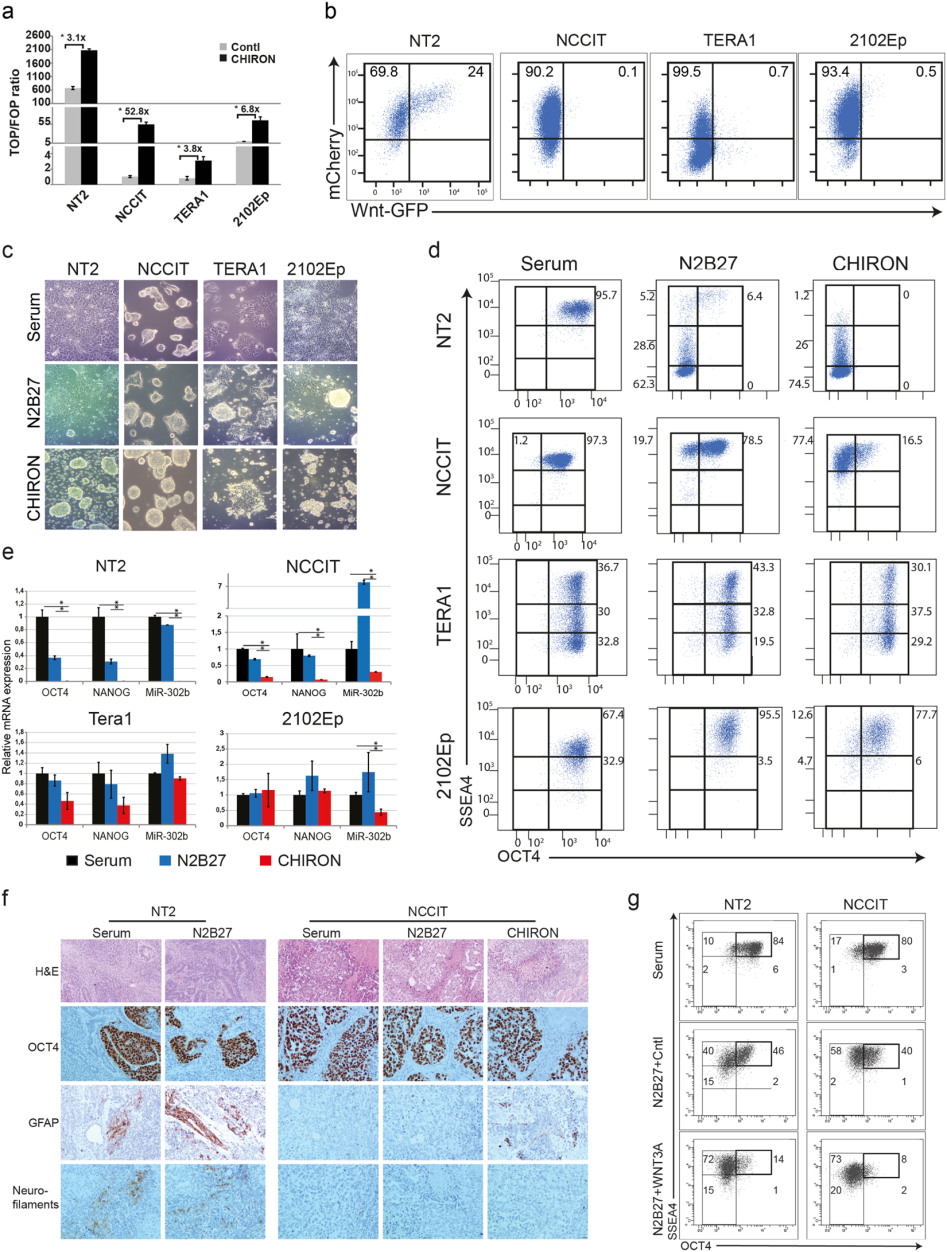
Intrinsic levels and short term activation of WNT signaling in different hEC lines. (**a**) Graph showing the β-catenin/TCF reporter assay in different EC lines before and after CHIRON-treatment. Different EC lines were treated with CHIRON or DMSO control for 48 h and the luciferase signal was measured for TOP and FOP plasmids using three biological triplicate. Numbers in the graph represent the fold changes of WNT induction. Bars represent mean ± SD, n = 3. Asterisk represent p-values < 0.05 that was calculated using two-tailed t-test. (**b**) EC cells were stably transfected with 7xTcf-eGFP lentiviral vector and the intrinsic WNT activity in different EC lines is represented by GFP signal. The ubiquitously expressed mCherry marks cells harboring the 7xTcf-eGFP plasmid. Numbers in the graph depict the percentage of positive-cells. (**c)** Light microscopy pictures showing the morphology of different EC lines cultured for 4 passages in N2B27 with or without CHIRON. Cells cultured in serum were used for comparison. (**d**) Flow cytometry analysis showing expression of the pluripotency-associated markers OCT4 and SSEA4 in hEC lines cultured for 4 passages in N2B27 or CHIRON-supplemented medium. Cells cultured in serum were used for comparison. Numbers in the graph represent the percent of positive cells in different regions. (**e**) qRT-PCR analysis of *OCT4*, *NANOG*, *SOX2* and *miR-302b* in EC lines cultured for 4-passages in N2B27 or CHIRON-supplemented medium. Cells cultured in serum were used for comparison. Bars represent n = 2 ± SEM. Asterisk represent p-values < 0.05 that was calculated using two-tailed t-test. (**f**) Teratoma samples were generated from NT2 and NCCIT cells cultured in serum, N2B27 or CHIRON-supplemented medium. NT2 cells cultured in CHIRON-supplemented medium failed to generate teratomas when injected into immunecompromised mice. Tissue sections were stained by H&E and were analyzed for multi-lineage differentiation using staining for GFAP (to mark the glial cell differentiation) and for Neurofilamnet (to mark the neural differentiation). OCT4 staining was employed to mark the undifferentiated EC cells within the teratomas. Note that teratomas generated from all NCCIT cultures were largely composed of undifferentiated OCT4-positive cells. NCCIT cells cultured in CHIRON-supplemented medium also displayed sparse and limited glial cell differentiation. (**g**) Induction of WNT signaling in NT2 and NCCIT cells using WNT3A conditioned medium. Cells were maintained in N2B27 supplemented with WNT3A (ratio of 1:3) or control medium for 5 days and were then employed in FACS analysis to evaluate SSEA4 and OCT4 expression.

We employed four representative hEC cell lines with distinct differentiation potential: NTera2/D1 (NT2) and NCCIT are pluripotent and have the potential to differentiate into somatic and extraembryonic cells (e.g. in response to retinoic acid treatment)^22,23^. In contrast TERA1 and 2102Ep are nullipotent EC cells and are defective in multi-lineage differentiation. To first measure the endogenous WNT signaling activity in the different EC cell lines, we employed the TOP-Flash WNT reporter assay^24^. We found that the pluripotent NT2 cells display the highest level of WNT signaling, whereas the NCCIT, TERA1 and 2102Ep EC cell lines show basal WNT activity (Fig. 1a).

To validate these results and to monitor the heterogeneity of WNT signaling at the cellular-level, we generated EC cell lines carrying a stably integrated TCF-eGFP WNT reporter construct^25^. The ubiquitously expressed mCherry was used to enrich for the lentiviral-transduced cells and GFP signal was employed to monitor WNT activity. In accordance with the above TOP-Flash reporter results, we found that NT2 cell line encompasses the largest subpopulation of GFP^+^WNT^+^ cells (24%), whereas the other EC cell lines have hardly detectable GFP-positive populations (ranging from 0.1% to 0.7%, Fig. 1b). Thus, with the exception of the NT2 cell line, the majority of examined EC lines display very low levels of WNT signaling.

### Short-term activation of WNT signaling induces distinct differentiation responses in hEC cells

To examine the effects of ectopic activation of WNT signaling, we cultured the different EC cell lines in the chemically-defined and serum-free N2B27 medium supplemented with CHIR99021 (CHIRON), an extremely specific GSK3-inhibitor commonly employed as a WNT activator^26^. TOP-Flash reporter assay, confirmed the induction of WNT-signaling upon CHIRON-treatment (Fig. 1a). Using flow cytometry analysis for the pluripotency associated markers OCT4 and SSEA4, we observed that NCCIT, TERA1 and 2102Ep cells display undifferentiated phenotype (OCT4^+^SSEA4^+^) when cultured in the control N2B27 medium similar to that observed in serum-supplemented medium (Fig. 1c,d). In contrast, only 6.4% of the NT2 cells retained high OCT4 and SSEA4 expression (Fig. 1d).

When cultured in CHIRON-supplemented medium, the pluripotent NT2 and NCCIT cells formed sphere-like structures notwithstanding the dramatic loss of OCT4 and SSEA4 markers in the vast majority of the cells (Fig. 1c). The latter was more pronounced in NT2 whereas a relatively small population of OCT4^+^SSEA4^+^ cells (16%) was retained in NCCIT line. In contrast to the pluripotent EC cells, the majority of the nullipotent 2102Ep and TERA1 cells maintained OCT4 and SSEA4 expression (67.1% and 83% respectively, Fig. 1c,d). In line with the flow cytometry results, qRT-PCR analysis for the pluripotency associated genes *OCT4*, *NANOG* and *miR-302b*^27,28^ confirmed the loss of pluripotency in CHIRON-treated NT2 and NCCIT cells and the maintenance of these markers in TERA1 and 2102Ep cells (Fig. 1e).

Teratoma formation represents the gold standard to assess the pluripotency potential of ES and EC cells^29^. To this aim, we injected subcutaneously the NT2 and NCCIT cells, cultured in serum, N2B27 or N2B27 + CHIRON medium, into NOD-SCID mice and assessed their differentiation. We have focused our attention on NT2 and NCCIT cells as they displayed morphological and gene expression changes in N2B27 or CHIRON cultures when compared to the serum-cultured controls. Of note, whereas NT2 cells cultured in serum or N2B27 medium formed tumors with multi-lineage differentiation (4/4), cells treated with CHIRON lost their teratoma-formation capacity and did not generate any palpable tumor (0/4). On the other hand, NCCIT cells cultured in serum, N2B27 or with CHIRON supplementation resulted in largely undifferentiated tumors that were mainly composed of OCT4-positive cells (4/4 in all three conditions, Fig. 1f). These results suggest that retention of an OCT4-positive population is associated with teratoma formation and perhaps once the CHIRON pressure is released *in vivo* these OCT4/SSEA4-positive cells contribute to teratomas formation. In NT2 cells cultured with CHIRON, loss of OCT4-positive population might explain why these cells failed to generate teratomas upon injected into immunocompromised mice.

To confirm that the effect of CHIRON is directly linked to the canonical WNT signaling, we activated the signaling pathway using WNT3A-conditioned medium^30^ in the responsive NT2 and NCCIT cells and we employed 2102Ep cells as control. In line with the observed effect of CHIRON, WNT3A-treatment resulted in loss of OCT4 expression in both NT2 and NCCIT cells (Fig. 1g**)** but had no effect on 2102Ep cells (data not shown). As expected, the effect of WNT3A-treatment was less pronounced when compared with CHIRON, reflecting the different modes of actions by the WNT3A-ligand and the CHIRON small molecule inhibitor; i.e. activation of WNT signaling by the upstream WNT3A-ligand versus the direct effect of CHIRON on the downstream GSK3-complex. We also observed that CHIRON- and to lesser extent WNT3A-treatment increased the percentage of cells in G1 phase of cell cycle in both NT2 and NCCIT cells suggesting that a high level of WNT signaling can lead to reduced cell proliferation in EC cells (Supplementary Fig. 1).

Taken together, the pluripotent NT2 and NCCIT cells rapidly differentiate in response to CHIRON treatment whereas the nullipotent TERA1 and 2102Ep cells are refractory to WNT activation.

### Long-term activation of WNT signaling induces culture adaptation in NCCIT and TERA1 cells

**Figure 2:**
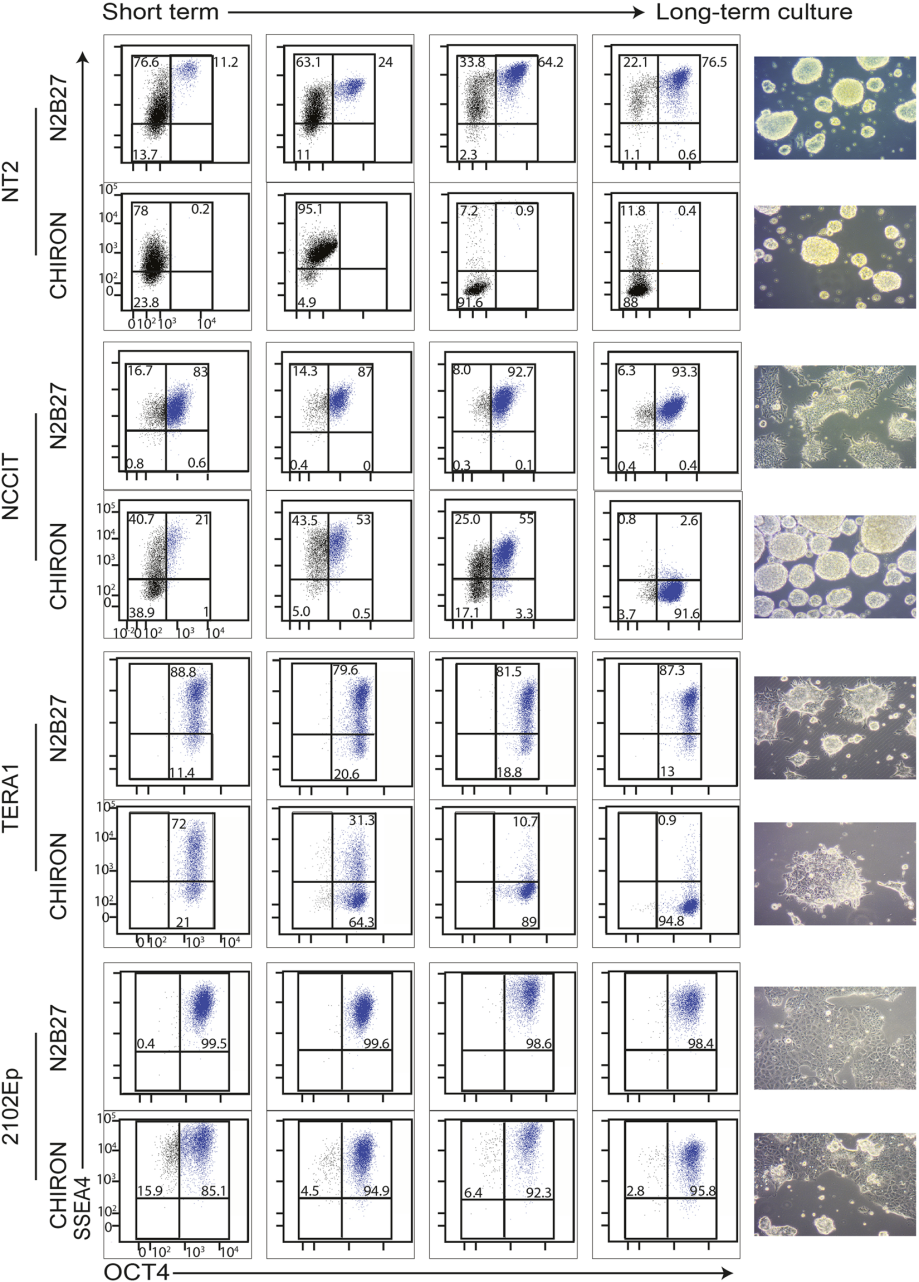
Time-course analysis of different hEC lines subjected to long term CHIRON treatment. Flow cytometry analysis for the pluripotency-associated markers OCT4 and SSEA4 in different EC lines cultured in N2B27 medium with or without CHIRON supplementation. Cells were collected every 5 passages and used in flow cytometry analysis. Early passage represents cells collected at passage 5 and long term culture represents cells collected at passage 20. The morphology of cells collected at passage 20 from N2B27 medium with or without CHIRON supplementation are shown in the figure.

In order to examine the long-term effects of WNT signaling, we analyzed the EC cells cultured in N2B27 or CHIRON-supplemented medium for over 20 passages. We collected cell-samples every 5 passages and analyzed the dynamics of OCT4/SSEA4 and in response to CHIRON-treatment. Similar to short-term cultures, high number of sphere-like structures was observed in CHIRON-supplemented medium, whereas cells cultured in the control N2B27 medium were largely monolayer (Fig. 2).

FACS analysis for OCT4 and SSEA4 revealed that NT2 cells maintain their differentiated OCT4^−^/SSEA4^−^ phenotype (99.6%) in long-term CHIRON-supplemented culture. In NCCIT cells, however, we observed a gradual adaptation to CHIRON-treatment; NCCIT cells displayed a gradual (re)gain of OCT4 and loss of SSEA4 markers, eventually resulting in a homogeneous OCT4^+^/SSEA4^−^ population (91.6%). TERA1 cells also displayed partial-adaptation to long-term CHIRON treatment; TERA1 cells remained OCT4-positive but similar to the NCCIT cells, lost the SSEA4 expression (95%). Of note, 2102Ep cells represented the most resistant EC line and remained OCT4/SSEA4-positive upon long-term CHIRON-treatment (96%, Fig. 2). Thus the different EC cell lines display a spectrum of responses to long term-CHIRON treatment resulting in OCT4^+^/SSEA4^−^ profiles in NCCIT and TERA1 cells.

### Long-term activation of WNT signaling induces SSEA4-to-SSEA1 switch and cisplatin-resistance

**Figure 3:**
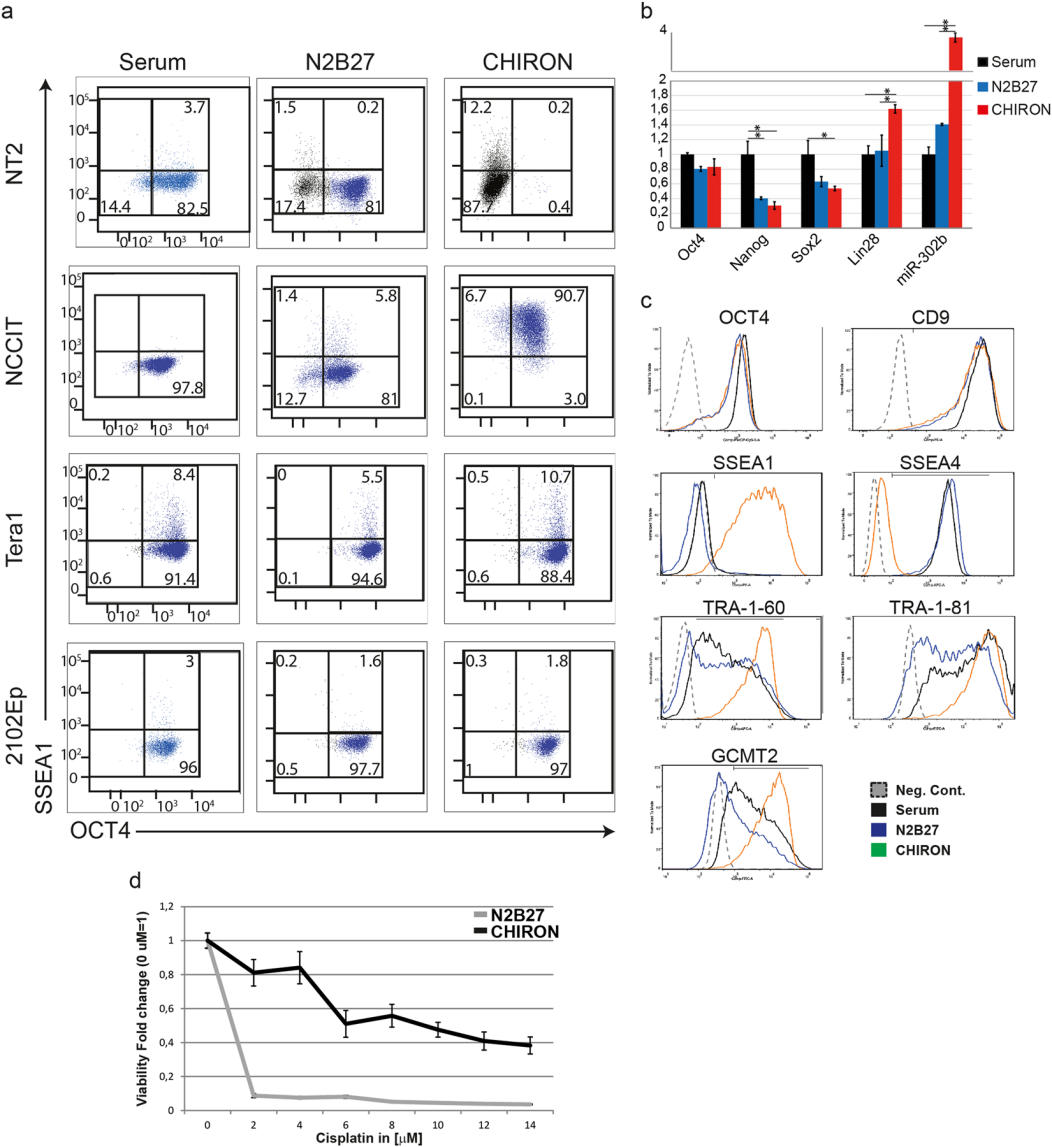
Long term WNT activation in NCCIT cells. (**a**) Flow cytometry analysis showing the expression of SSEA1 and OCT4 in different EC lines cultured for 20 passages in N2B27 or CHIRON-supplemented medium. Cells cultured in serum were used for comparison. (**b**) qRT-PCR analysis of the pluripotency markers *OCT4*, *NANOG*, *SOX2*, *LIN28* and *miR-302b* in NCCIT cells cultured for over 20 passages in N2B27 or CHIRON-supplemented medium. Bars represent n = 2 ± SEM. Asterisk represent p-values < 0.05 that was calculated using two-tailed t-test. (**c**) Flow cytometry analysis for different EC-specific markers in NCCIT cells cultured for over 20 passages in N2B27 with or without CHIRON supplementation. Serum cultured cells were used as control. (**d**) Graph showing the cellular viability of cisplatin treated cells. NCCIT cells were cultured for 20 passages in N2B27 or CHIRON-supplemented medium and were then treated with different concentrations of cisplatin for three days. Cellular viability was measured using WST1 assay and fold change ratios compared to non-treated cells are depicted in the histogram n = 3 ± SD.

In human ES/EC cells, SSEA4 and OCT4 expression is commonly employed to mark the undifferentiated cells whereas more differentiated cells lose both SSEA4 and OCT4 and acquire SSEA1 expression^31–35^. Undifferentiated mouse ES/EC cells, in contrast, exhibit both SSEA1 and OCT4 expression and are negative for SSEA4. The loss of SSEA4 and maintenance of OCT4 in long-term CHIRON-treated NCCIT and TERA1 cells triggered us to investigate whether SSEA4 is replaced by SSEA1 antigen similar to the mouse ES/EC cells. We also employed the NT2 and 2102Ep as negative and positive controls, respectively. In NT2 cells, we found that SSEA1 was confined to the OCT4/SSEA4-negative population, confirming the differentiated state of these cells (Fig. 3a). As expected, the 2102Ep cells did not show any SSEA1 expression (1.8%). Interestingly, we found that all the NCCIT cells cultured in CHIRON-supplemented medium gained SSEA1 expression (90.7%), generating a new state of EC cells earmarked by OCT4^+^/SSEA4^−^/SSEA1^+^ that is reminiscent of mouse ES cells (Fig. 3a). These NCCIT cells also maintained high levels of the pluripotency markers *NANOG*, *SOX2* and *miR-302b* (Fig. 3b). To a lesser extent, a gain of SSEA1 expression was also observed in CHIRON-treated TERA1 cells (10.7%) and this expression was confined to the SSEA4-negative but OCT4-positive population. We also observed SSEA1 expression in TERA1 control cells (cultured in serum or in N2B27 medium), however, they are SSEA4-positive, i.e. indicative that they may represent the transition process gradually loosing SSEA4 and gaining SSEA1 expression.

To further characterize the OCT4/SSEA1-positive population, we examined the expression of other previously described cell surface markers for undifferentiated EC cells^31,36^. We found that despite the SSEA4-to-SSEA1 profile switch, the long-term CHIRON-treated NCCIT cells do express other pluripotency associated antigens including CD9, TRA-1-60, TRA-1-81 and GCMT2 confirming the undifferentiated state of the cells (Fig. 3c). Thus continuous exposure of NCCIT and TERA1 cells to CHIRON, led to a cell state marked by OCT4 and SSEA1 and lack of SSEA4 expression.

Finally, we asked whether the SSEA4-to-SSEA1 switch within the OCT4-positive population is of functional relevance. (T)GCTs display unique chemo-sensitivity to cisplatin treatment, mainly associated with the undifferentiated state of the cells^37,38^. We therefore examined the NCCIT cell line as it was composed by relatively pure populations of OCT4^+^/SSEA1^−^/SSEA4^+^ and OCT4^+^/SSEA1^+^/SSEA4^−^ cells when cultured in N2B27 in the absence or presence of CHIRON, respectively. We did not examine the TERA1 line in this experiment as TERA1 cells displayed only ~10% SSEA1-positive population in CHIRON culture and a small percentage of SSEA1-positive cells were also present in serum and N2B27 controls. For this analysis, we examined the cisplatin-sensitivity in NCCIT cells and found that whereas N2B27-cultured cells are highly sensitive to cisplatin treatment, CHIRON-treated cells display higher cisplatin-resistance. These results suggest that WNT induction conferred a chemo-resistance in the cells despite the expression of the pluripotency markers and the partially undifferentiated state of the cells (Fig. 3d).

## Discussion

In this study, we show that short-term activation of WNT signaling induces a differentiation response in the pluripotent NT2 and NCCIT cells whereas it has a very limited effect on the nullipotent TERA1 and 2102Ep cell lines. Our data corroborate and extend the previously described role of WNT signaling in hESCs, where high level of WNT signaling induces differentiation and loss of pluripotency^14,15,18^. In agreement with our finding, OCT4 was shown to repress β-catenin and to maintain low level of WNT signaling in the undifferentiated cells^18^. Furthermore, WNT signaling activity increases during differentiation of EC cells^39^ and overexpression of *Axin*, a negative regulator of WNT signaling, blocks the differentiation induced by retinoic acid in EC cells^40^. Taken together, in contrast to mouse ES/EC cells, high levels of WNT signaling induces loss of pluripotency in hES and EC cells and may induce transition of EC into more differentiated tumor types such as teratoma and YST.

By measuring the endogenous WNT activity in different and frequently used EC cell lines, we found that NT2 cells display the highest signaling levels. By employing reporters of endogenous WNT activity, we show that this high levels of WNT signaling is mainly observed in only 25% of the NT2 cells. Further, although both NT2 and NCCIT cells are responsive to WNT induction, our data suggest that NT2 cells display a stronger differentiation response (i.e. upon transfer from serum to N2B27 medium or treatment with CHIRON) when compared to NCCIT cells. In this regard, the high level of endogenous WNT signaling might underlie both the basal differentiation observed in control NT2-cultures and the strong differentiation response upon ectopic WNT activation.

We report the co-expression of OCT4 and SSEA1 and the lack of SSEA4 in undifferentiated hEC cells (NCCIT and TERA1) upon long-term WNT-signaling activation. In human embryo, SSEA4 is restricted to the inner cell mass (ICM) cells, whereas SSEA1 is detected in the trophectoderm while it is absent in the ICM^41^. Further, immunohistochemistry and flow cytometry analysis indicate that SSEA1 is not expressed in hES/EC cells but is detected upon differentiation^23,36,42^. Our findings suggest the intermediate state of OCT4^+^/SSEA1^+^ that can be obtained using long term activation of WNT signaling. This observation might be of relevance for chemotherapy resistance in TGCC in view of our observation according to which the OCT4^+^/SSEA1^+^/SSEA4^−^ cells are more resistant to cisplatin treatment. The detection of a novel OCT4^+^/SSEA1^+^/SSEA4^−^ population of EC cells is also of interest for the generation of different pluripotency states that can shed light on the genesis of TGCTs and the reprogramming of hESCs to a naïve-state pluripotency (for review see^43^). In this regard, hEC cells might provide a suitable model as they display basic culture requirements and harbor genetic abnormalities such as gain of 12p^19^ (spanning NANOG and GDF3 loci) or SOX2 amplification^44^ that can bypass the ectopic over-expression of the pluripotency factors.

## Materials and Methods

### Cell culture

The, NTera2/D1 (NT2) and 2012EP cells were kindly provided by Dr. Peter Andrews, University of Sheffield, UK. NCCIT was a gift from Dr. Ivan Damjanov, University of Kansas, USA. TERA1 and TCam-2 cells were obtained from Dr. Sohei Kitazawa, Kobe University, Japan). Cells were maintained in DMEM (Gibco) supplemented with 10% fetal calf serum (FCS, Gibco), L-glutamine (2 nM, Gibco), Na-Pyruvate (1 mM, Gibco) and 1 mM penicillin/streptomycin. Cells were trypsinized or scraped (in case of NT2) and passaged every 2–3 days. For the Serum Free culture, cells were plated in N2B27 medium consisting of DMEM/F12:Neurobasal medium (1:1, Gibco) supplemented with N2 and B27 (Gibco). N2B27 medium was then supplemented with CHIR99021 (3 μM, Stemgent). The medium was refreshed every 2–3 days and cells were passaged at 1:2 to 1:8 splitting ratio depending on the cell line.

### RNA extraction, cDNA synthesis and qRT-PCR

RNA was isolated using the RNeasy Mini Kit (QIAGEN) or Trizol (Invitrogen) and the isolated RNA was used in a DNase treatment step (Ambion) to remove the residual genomic DNA. cDNA was synthesized using 1 μg RNA and the RevertAid™ H Minus First Strand cDNA Synthesis Kit (Thermo). microRNA expression analysis was performed using 40 ng of total RNA and employed in cDNA synthesis reaction using TaqMan^TM^ MicroRNA Reverse Transcription kit (ABI). The Delta-Ct method was used to quantify the relative gene expressions. qRT-PCR analysis of the selected genes were performed using Fast SYBR® Green Master Mix (ABI) and the primers listed in (Supplementary Table S1).

### Teratoma assay

2–4 million cells grown in different conditions were injected in Matrigel (BD Biosciences) and subcutaneously in NOD-SCID mice. Teratomas were collected after 3–4 weeks.

### Immunohistochemistry analysis

Teratomas samples were fixed in PFA (4%) and embedded in paraffin. 5 µm sections were mounted on slides stained by H&E for routine histology. Antibodies for IHC analysis included: mouse 2H3 against Neurofilaments (1∶50, Developmental Studies Hybridoma Bank); rabbit anti-GFAP (1∶5000, Z0334, DAKO,); mouse anti-Oct3/4 (1∶100, sc-5279, Santa Cruz). Signal detection was performed using HRP-conjugated mouse and rabbit Envision kits (Dako).

### Flow Cytometry analysis

1 × 10^6^ cells were stained for PE-conjugated anti-SSEA1 (BD Biosciences 560142) and Alexa Flour 647 conjugated anti-SSEA4 (BD Biosciences 560477) for 30 minutes following by fixation in 4% PFA for 20 minutes at room temperature. Cells were then permeabilized with a BD-permeabilization buffer (BD Biosciences 51-2091KZ) for 10 minutes and stained subsequently with PerCP-CY5.5-conjugated anti-OCT4 antibody (BD Biosciences 560477) for 30 minutes at room temperature. As control, non-stained sample or cells stained with isotype controls were used. Flow cytometric analysis was performed with a BD FACSAria III.

### Lentiviral production

pLenti 7xTcf-eGFP vector^25^ was purchased from Addgene and lentiviral particles were produced in HEK-293 using the pMD2.G envelope plasmid and the psPAX2 packaging plasmid as described previously^45^. The medium was replaced the day after and the conditioned medium was collected after 2 and 4 days and filtered through a 0.45 µm filter. The virus-containing medium was diluted 1:2 with normal medium and together with polybrene (to enhance the viral transduction) was added to EC cells. MCherry–positive cells were FACS sorted subsequently to enrich for the transduced cells.

### WNT reporter assay

For β-catenin/TCF reporter assay, cells were plated in 24-well plate one day before transfection and transfected with 250 ng TOP-Flash or FOP-Flash reporter plasmids^24^ using FuGENE HD transfection reagent (Promega). To normalize for transfection efficiency, 20 ng SV40-Renilla Luciferase plasmid was employed. luciferase activity was measured 48 h post-transfection using Dual–Luciferase Reporter Assay System (Promega). For WNT stimulation, CHIRON (Stemgent) or DMSO (as control) was added to cells at 3 μM concentration and for 48 h.

### Cisplatin treatment and WST1 assay

NCCIT cells grown in N2B27 medium supplemented with or without CHIRON for over 20 passages, were employed in chemoresistance assay. Briefly, cells were plated at similar density in N2B27 medium supplemented with or without CHIRON and cisplatin (Teva Pharmachemie) was added at different concentrations for 3 days. Cells were then employed in WST1 assay (Roche) according to manufacturer’s instructions to assess the cellular proliferation and viability. Briefly, cells were incubated with WST1 reagent for 3 h and the signal was measured afterwards using the scanning multi-well spectrophotometer (ELISA reader, Bio-Rad) at 490 nm and 595 nm.

### Cell cycle analysis

Cells were fixed in 70% ethanol for 1 h on ice and were stained with Propidium Iodide solution (10 ug/ml PI [Sigma, P4170] and 0.2 mg/mL RNase A in PBS) over night at 4 °C. 15,000 were analyzed using BD FACSAria III.

### Statistics and reproducibility

Microsoft Excel and GraphPad Prism6 was used for statistical analyses. Error bars, P-values and statistical tests are reported in the figure legends. Statistical tests include unpaired two-tailed Student’s t-test.

## Supplementary information

Supplementary info

## Data Availability

All data are available upon reasonable request from the corresponding author.
