# Supplementary material for: Ectopic activation of WNT signaling in human embryonal carcinoma cells and its effects in short- and long-term in vitro culture
Source: Sci Rep. 2019 Aug 15;9:11928. doi: 10.1038/s41598-019-48396-7 (PMC6695393; doi:10.1038/s41598-019-48396-7)
Supplement: Supplementary file 1 — Supplementary info [file 41598_2019_48396_MOESM1_ESM.pdf]

# Supplementary Information

## Ectopic activation of WNT signaling in human embryonal carcinoma cells and its effects in short- and long-term *in vitro* culture

Yaser Atlasi, Rebecca T. van Dorsten, Andrea Sacchetti, Rosalie Joosten, J. Wolter Oosterhuis, Leendert H.J. Looijenga and Riccardo Fodde

**Supplementary Figure S1.**

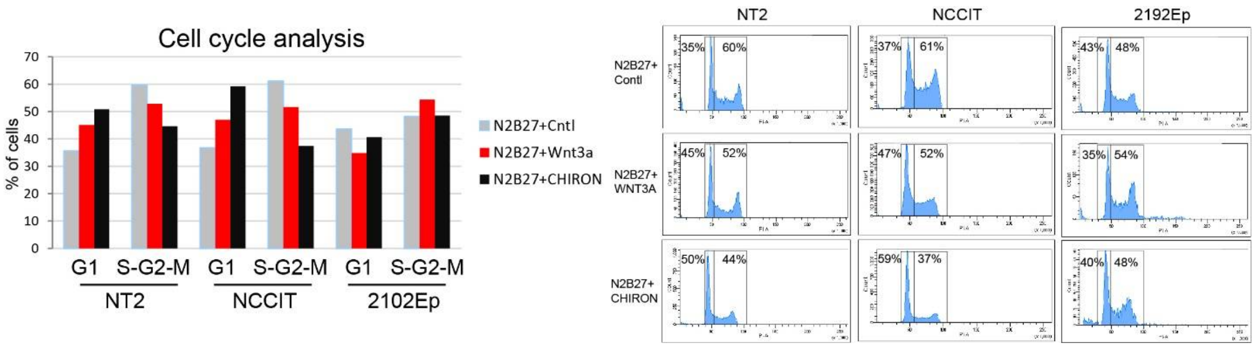


**Supplementary Figure 1: Cell cycle analysis in EC cells upon activation of WNT signaling**
Graph (left) showing the increased percentage of cells in G1 phases of the cell cycle in NT2 and NCCIT cells. 2102Ep cells were used as control. Histograms (right) showing the Propidium Iodide staining (PI) in NT2 and NCCIT cells. Numbers in the graphs depict the percentage of cells in G1 or S-G2-M phases that were used in the left graph. Cells were treated with WNT3A-conditioned medium, control medium or CHIRON for 2 passages (5 days) and were employed in PI-staining.

**Supplementary Table 1**: **Sequences of the employed qPCR primers.**

| Gene | Forward | Reverse |
|---|---|---|
| h-NANOG | CGGAGACTGTCTCTCCTCTTC | GGTAGGTGCGAGGCCTTCTGC |
| hOct4 | AGTGAGAGGCAACCTGGAGA | ACACTCGGACCACATCCTTC |
| hSox2 | TGGACAGTTACGCGCACAT | CGAGTAGGACATGCTGTAGGT |
| h-LIN28 | GGCATCTGTAAGTGGTTCAAC | TACAGAATACTCCACCAGGTC |
| h-HPRT1 | CGTGGGGTCCTTTTCACCAGCAAG | AATTATGGACAGGACTGAACGTC |
